# Role of indoleamine 2,3-dioxygenase in testicular immune-privilege

**DOI:** 10.1038/s41598-019-52192-8

**Published:** 2019-11-04

**Authors:** Gisela S. Gualdoni, Patricia V. Jacobo, Cristian M. Sobarzo, Cecilia V. Pérez, María E. Matzkin, Christian Höcht, Mónica B. Frungieri, Marcelo Hill, Ignacio Anegon, Livia Lustig, Vanesa A. Guazzone

**Affiliations:** 1 Universidad de Buenos Aires (UBA), Facultad de Medicina, Departamento de Biología Celular e Histología/Unidad Académica II., Ciudad Autónoma de Buenos Aires, C1121ABG Argentina; 2 Consejo Nacional de Investigaciones Científicas y Técnicas (CONICET), Universidad de Buenos Aires (UBA), Instituto de Investigaciones Biomédicas (INBIOMED), Facultad de Medicina, Ciudad Autónoma de Buenos Aires, C1121ABG Argentina; 3 Instituto de Biología y Medicina Experimental (IBYME), CONICET, Ciudad Autónoma de Buenos Aires, C1428ADN Argentina; 4 Cátedra de Farmacología. Facultad de Farmacia y Bioquímica, UBA, Ciudad Autónoma de Buenos Aires, C1121ABG Argentina; 5 Laboratory of Immunoregulation and Inflammation, Institut Pasteur de Montevideo, 11400 Montevideo, Uruguay; 6 Immunobiology Department, Faculty of Medicine, University of the Republic, 11800 Montevideo, Uruguay; 7 Inserm, Université de Nantes, Centre de Recherche en Transplantation et Immunologie, Nantes, France INSERM UMR 1064 France

**Keywords:** Autoimmunity, Autoimmunity, Chronic inflammation, Chronic inflammation

## Abstract

Male meiotic germ cell including the spermatozoa represent a great challenge to the immune system, as they appear long after the establishment of normal immune tolerance mechanisms. The capacity of the testes to tolerate autoantigenic germ cells as well as survival of allogeneic organ engrafted in the testicular interstitium have led to consider the testis an immunologically privileged site. Disruption of this immune privilege following trauma, tumor, or autoimmune orchitis often results in male infertility. Strong evidence indicates that indoleamine 2,3-dioxygenase (IDO) has been implicated in fetal and allograft tolerance, tumor immune resistance, and regulation of autoimmune diseases. IDO and tryptophan 2,3-dioxygenase (TDO) catalyze the same rate-limiting step of tryptophan metabolism along a common pathway, which leads to tryptophan starvation and generation of catabolites collectively known as kynurenines. However, the relevance of tryptophan metabolism in testis pathophysiology has not yet been explored. Here we assessed the *in vivo* role of IDO/TDO in experimental autoimmune orchitis (EAO), a model of autoimmune testicular inflammation and immunologically impaired spermatogenesis. EAO was induced in adult Wistar rats with testicular homogenate and adjuvants. Control (C) rats injected with saline and adjuvants and normal untreated rats (N) were also studied. mRNA expression of IDO decreased in whole testes and in isolated Sertoli cells during EAO. TDO and IDO localization and level of expression in the testis were analyzed by immunostaining and Western blot. TDO is expressed in granulomas from EAO rats, and similar protein levels were observed in N, C, and EAO groups. IDO was detected in mononuclear and endothelial cells and reduced IDO expression was detected in EAO group compared to N and C rats. This phenomenon was concomitant with a significant reduction of IDO activity in EAO testis measured by tryptophan and kynurenine concentrations (HPLC). Finally, *in vivo* inhibition of IDO with 1-methyl-tryptophan increased severity of the disease, demonstrating down regulation of IDO-based tolerance when testicular immune regulation was disrupted. We present evidence that an IDO-based mechanism is involved in testicular immune privilege.

## Introduction

The testis is considered an immunoprivileged organ since it tolerates germ cell antigens appeared during the pubertal period, an event developing long after establishment of immunocompetence. Also, the testis itself induces tolerance after its transplantation to an allogenic recipient, and is conversely able to accept foreign cells without triggering immune rejection^1^.

Multiple mechanisms are involved in testis immune privilege: a blood testis barrier modulates antigen and antibody traffic inside and outside seminiferous tubules (ST), secretion of immunosuppressor molecules (e.g. transforming growth factor [TGF] β, interferon [IFN] α, β) by somatic and germ cells and the presence of local and systemic regulatory T (Treg) cells induce a testicular immunosuppressive microenvironment^2,3^. Tung *et al*.^4^ have shown that, in contrast with a previous paradigm, only some murine meiotic germ cell antigens are sequestered and are not tolerogenic. In contrast, non-sequestered germ cell antigens egress from normal tubules and interact with circulating antibodies, forming immune complexes at the wall of ST. Non-sequestered germ cell antigens originated from fragments discarded by spermatids during spermiogenesis maintain physiological tolerance dependent on Treg. Autoimmune responses to spermatic antigens occur in human infertility and after vasectomy as well as in experimental models of autoimmune orchitis. Moreover, many germ cell antigens are also expressed as cancer antigens in human testicular neoplasia.

Two decades ago Sertoli cells (SC) were characterized as immunosuppressor cells^5,6^ providing a protective microenvironment for some grafts in co-transplantation experiments^7,8^. Fallarino *et al*.^9^ demonstrated that isolated neonatal porcine SC prevent and revert diabetes in non obese diabetic mice, being cell or insulin therapy unnecessary. This result was explained by the restitution of systemic immune tolerance dependent on efficient tryptophan metabolism in the xenografts.

Indoleamine-2,3-dioxygenase (IDO) and tryptophan 2,3-dioxygenase (TDO) are intracellular heme-containing enzymes that catalyze oxidative cleavage of L-tryptophan to produce kynurenines. Depletion of L-tryptophan and production of kynurenine can modulate adaptive immune response^10,11^.

In dendritic cell (DCs), IDO expression is normally maintained at low basal levels but can be rapidly induced by IFNγ^12,13^. The IFNγ–IDO mechanism limits microbial growth and reduces injurious hyperinflammatory responses in the host^14^. The TGFβ–IDO axis has a primary role in the generation and maintenance of Treg cells^15–17^. Thus, IDO has a chief role in peripheral generation of Treg cells both in physiology and pathophysiology. IDO activity contributes to maternal tolerance in pregnancy^18^, controls allograft rejection^19,20^, and protects against autoimmunity such as, trinitrobenzene sulfonic acid colitis^21^, rheumatoid arthritis^22^, granulomatous diseases^14,23^ and allergy^24,25^.

Although several reviews mention IDO as a molecule involved in testis immune privilege, the only evidence supporting this concept is the expression of IDO in porcine SC and the ability of these cells to restore immune tolerance in NOD mice^9^. Functional studies are therefore lacking to show a potential physiological and/or pathological role for IDO-mediated immunoregulation in testis.

With the aim of contributing to understanding of the role of IDO/TDO in testis immunoprivilege in physiological and inflammatory conditions, we evaluated the expression, distribution, and activity of this molecule in the testis of untreated normal (N), control (C), and rats with experimental autoimmune orchitis induced by active immunization with sperm antigens and adjuvants. We studied a potential functional role for IDO *in vivo* by analyzing the incidence and severity of orchitis in immunized rats that received an IDO inhibitor.

## Materials and Methods

### Animals

Adult male inbred Wistar rats aged 50–70 days were purchased from Bioterio Central Facultad de Farmacia y Bioquímica (Buenos Aires, Argentina). Animals were kept at 22 °C with 14 h light, 10 h dark schedule and fed standard food pellets and water *ad libitum*. The use of rats followed NIH guidelines for care and use of experimental animals, also approved by local committees (Comité Institucional para el Cuidado y Uso de Animales de Laboratorio (CICUAL)-Facultad de Medicina).

### Induction of experimental autoimmune orchitis (EAO)

Rats were actively immunized with syngeneic testicular homogenate (TH) emulsified with complete Freund’s adjuvant (CFA; Sigma-Aldrich, St Louis, MO, USA) into the hind footpads and in different sites of flanks and near the neck area (subcutaneously) as described previously^26^. The first two immunizations were followed by an intravenous injection of 10^10^ inactivated *Bordetella pertussis* (Bp) bacteria (strain 10536; kindly provided by Instituto Malbrán, Buenos Aires, Argentina), whereas the third was followed by an intraperitoneal injection of Bp at a concentration of 5 × 10^9^. Rats of control (C) group received CFA and Bp, but no TH, otherwise following the same scheme. Normal (N) untreated rats were also studied. Rats were killed 50 days after the first immunization. Testes, epididymis and popliteal, inguinal, renal and iliac lymph nodes (LN) were removed, weighed, and processed as described below. Only rats developing orchitis after 50 days were studied (EAO rats).

### Histopathology

Histopathology of testis and epididymis was evaluated in paraffin-embedded Bouin’s-fixed sections obtained from three different levels and stained with hematoxylin-eosin. To evaluate the severity of EAO, we used a score described previously^27^. This score was graded by evaluation of (a) percentage of ST with impairment of spermatogenesis, (b) degree of germ cell sloughing and (c) testicular/body weight ratio (T/Bw). Maximum EAO score is 10. Animals with a score equal to 0 were considered free of orchitis. Epididymal pathology was graded by evaluation of caput, corpus, and cauda inflammation and sperm depletion using an established score^28^. Epididymal inflammation (epididymitis): 1–5 and sperm depletion: 0–2 represent the range of incremental inflammation and decrement of sperm, respectively.

### Immunohistochemistry

Rabbit polyclonal antibodies anti-IDO that recognize at least rat and human IDO1^29^ or anti-TDO (GeneTex, Irvine, CA, USA) were used to detect expression and localization of IDO and TDO proteins in acetone-fixed frozen testis and LN sections (7 µm thick). Sections were incubated with 5% normal goat serum, 0.03% Triton X-100 containing 4% bovine serum albumin (BSA) for IDO or with 5% skim milk and 0.01% Triton X-100 for TDO 30 min at room temperature, and treated with avidin/biotin blocking solution (Vector Lab., Burlingame, CA, USA) followed by overnight incubation with the primary antibody IDO (1/500) or TDO (1/25) at 4 °C in a humidified chamber. A biotinylated goat anti-rabbit IgG (1/250, Vector Lab.) was used as secondary antibody. Endogenous peroxidase activity was blocked by treatment with 0.01% H_2_O_2_ in methanol for 30 min. The reaction was amplified with the Vectastain Elite ABC Kit (Vector Lab.), and the reaction product was visualized by the addition of diaminobenzidine substrate (Vector Lab.). Sections were counterstained with hematoxylin. Negative controls were obtained by incubating sections with phosphate-buffered saline (PBS) instead of primary antibodies.

Co-expression of IDO1 and ED1, ED2 or CD31 was detected in methanol-fixed cryostat testis sections by indirect immunofluorescence. Mouse monoclonal antibodies anti-ED1 (1/30, BD Pharmingen, San Diego, CA, USA) or anti-ED2 (1/50, BD Pharmingen) recognize a cytoplasmic antigen in rat monocytes, macrophages, and dendritic cells or membrane antigen of tissue macrophages, respectively. Mouse CD31 antibody (1/25, Genway Biotech Inc., San Diego, CA, USA) recognizes endothelial cells. Sections were incubated with 5% normal donkey serum followed by 120 min incubation with ED1 or ED2 antibodies or 5% normal horse serum followed by overnight incubation with CD31 antibody. Anti-cyanine 3 conjugated mouse IgG (1/500, Merck, Darmstadt, Germany) was used as secondary antibody. Then, sections were blocked as described above, followed by overnight incubation with IDO1 antibody. Anti-fluorescein isothiocyanate conjugated rabbit IgG (1/50, Vector Lab.) was used as secondary antibody. Sections were counterstained with 4′,6-diamidine-2-phenylindole (DAPI). Negative controls were obtained by incubating sections with PBS instead of primary antibodies.

### qRT-PCR

Total RNA was isolated from testes of adult rats using TRIzol reagent (Life Technologies, Carlsbad, CA, USA) according to manufacturer’s instructions. The qRT-PCR assays were performed using oligonucleotide primers for *18S* rRNA (5′-ACACGGACAGGATTGACAGATT; 5′-CGTTCGTTATCGGAATTAACCA) and rat *IDO* (5′-AGCACTGGAGAAGGCACTG and 5′- CGTGGAAAAAGGTGTCTGG). 18S rRNA was chosen as housekeeping gene. Reactions were conducted using FastStart Universal SYBR Green Master Mix (Roche Diagnostic, Mannheim, Germany) and the CFX-96 touch sequence detector System (Bio-Rad, Hercules, CA, USA). Reaction conditions were as follows: 10 min at 95 °C (one cycle), followed by 40 cycles of 15 s at 95 °C, 30 s at 55 °C and 1 min at 60 °C for 18S, or by 40 cycles of 15 s at 95 °C and 1 min at 60 °C for IDO. Following the mathematical model of Pfaffl^30^, the fold change of mRNA IDO expression was determined for each sample as described previously^31^.

### Immunohistochemical analysis followed by laser capture microdissection and RT-PCR

A goat polyclonal anti-vimentin antibody (1/10, Santa Cruz Biotechnology, Dallas, Texas, USA) was used to identify Sertoli cells in testes paraffin embedded sections. An immunoperoxidase technique using the avidin-biotin system was applied as described above. Subsequently, laser capture microdissection (LCM) was performed. Laser energy of the Applied Biosystems (Bedford, MA, USA) Arcturus LCM equipment was used to circumscribe vimentin immunopositive Sertoli cells as previously described by Rossi^31^. Microdissected samples containing immunopositive Sertoli cells were used for RT-PCR. Total RNA was isolated using TRIzol reagent (Life Technologies) according to the manufacturer’s instructions. Purity of total RNA isolated was assessed using specific markers of germ cells (VASA) and Sertoli cells (FSH-receptor). The following oligonucleotide primers were used for amplification of IDO (first set 5′-GCAGTAGAGCATCAAGACC and 5′- CGTGGAAAAAGGTGTCTGG, hemi-nested set 5′-AGCACTGGAGAAGGCACTG and 5′-CGTGGAAAAAGGTGTCTGG), VASA (5′-CCAAGAGAGGCGGCTATCGAGATG and 5′-AGAACCAAAAAGGCCAACCAGTGCG), and FSH-receptor (5′-CCTTGCTCCTGGTCTCCTTG and 5- GGAAGACCCTGTCAGAGC). 18S amplification was performed using the oligonucleotide primers previously described in qRT-PCR section. PCR conditions were 95 °C for 5 min, followed by 30–45 cycles of 94 °C for 1 min, 60 °C (annealing temperature) for 1 min, 72 °C for 1 min, and a final incubation at 72 °C for 5 min. PCR products were separated on 2% agarose gels and visualized with ethidium bromide.

### Western blots

Expression of IDO and TDO proteins were evaluated in testis, in a pool of renal and iliac LN (testicular draining LN; TLN) and a pool of inguinal and popliteal LN (LN draining the site of immunization, ILN). ST were mechanically separated from testicular interstitial cell (IC) and homogenized in ice-cold lysis buffer as described previously^32^. LN were cut with scissors and incubated with the lysis buffer. 200 μg of proteins were resolved in a 10% SDS-polyacrylamide gel electrophoresis, electroblotted at 66 mA for 18 h onto PVDF membranes (Bio-Rad Laboratories) and transfer was monitored by Ponceau S staining. Membranes were blocked with 5% non-fat dry milk in TBS containing 0.1% Tween 20 for 1 h. Blots were probed overnight with a rabbit anti-IDO^29^ (1/100), rabbit anti-IDO2 (1/100) or anti-TDO (1/500, GeneTex) polyclonal antibody followed by anti-actin (1/6000; Sigma-Aldrich) polyclonal antibody. Blots were washed and incubated with a biotinylated goat anti-rabbit IgG (1/3000; Vector Lab.) followed by streptavidin-horseradish peroxidase conjugates (Chemicon International Inc, Millipore Co., Billerica, MA, USA). Proteins were visualized by enhanced chemiluminescence and images were captured using GeneSnap software (7.08.01 version) and analyzed with Gene Tools software (4.01.02 version) from SynGene (Synoptics Ltd, Frederick, MD, USA).

### IDO/TDO activity assay

To detect IDO/TDO activity decapsulated testes, TLN and ILN were cut with scissors. Samples were incubated with lysis buffer as described above and no protease inhibitors were added. Protein precipitation was performed by adding 70% perchloric acid to the supernatant. The suspension was left on ice for 15 min, and then centrifuged at 22,000 g for 10 min. The pellet was discarded and 100 μl of the supernatant was analyzed by HPLC. Kynurenine (Kyn) release and tryptophan (Trp) degradation were measured. Standard curves were generated with L-Kyn and L-Trp (Sigma-Aldrich) in the same solvent. The chromatographic system was equipped with a Phenomenex Luna 5 μm, C18, 250 mm × 4.60 mm column and an ultraviolet detector (UVIS 204, Linear Instruments, Reno, USA). The wavelength was set at 360 nm for Kyn and at 278 nm for Trp. The mobile phase composed of acetate buffer (15 mM, pH 4) (92%) and acetonitrile (8%) was pumped at a flow rate of 1.0 ml min^−1^. Results were shown as the Kyn/Trp ratio.

### Administration of 1- Methyl-Tryptophan (1-MT)

Based on Hou and Muller^33^, we used IDO specific inhibitor 1- D- MT (Sigma-Aldrich). It was administered 5 days per week by oral gavage at 10 mg/dose (0.1 ml/rat) during the immunization period (from day 0 to day 28) to rats immunized with TH and adjuvants. Another group of immunized rats received 0.1 ml of diluent solution (0.5% Methylcellulose, 0.5% Tween 80) (vehicle). Animals were killed 50 days after first immunization. Body weight was determined and testes and epididymis were removed and processed for histopathology.

### Delayed type hypersensitivity (DTH)

DTH was measured by a footpad swelling test performed at the end of the *in vivo* assay as previously described^34^.

### Statistical analysis

Results are expressed as mean ± SEM. Comparisons between groups were assessed by the non-parametric Mann-Whitney *U* test or Kruskal–Wallis One-Way ANOVA. *P* values less than 0.05 were considered significant.

## Results

### Histopathology

As described previously^26^ rats immunized with TH and adjuvants developed autoimmune orchitis characterized by mild interstitial infiltration of lymphomononuclear cells intermingled with Leydig cells and multiple foci of damaged ST with different degrees of germ cell sloughing. Some rats with severe orchitis presented severe germ cell sloughing in most ST in which only SC and basal germ cells (mainly spermatogonia and few pre leptotene spermatocytes) remained attached to the tubular wall and granulomae.

### mRNA expression of IDO decreased in whole testes and isolated SC during EAO

**Figure 1:**
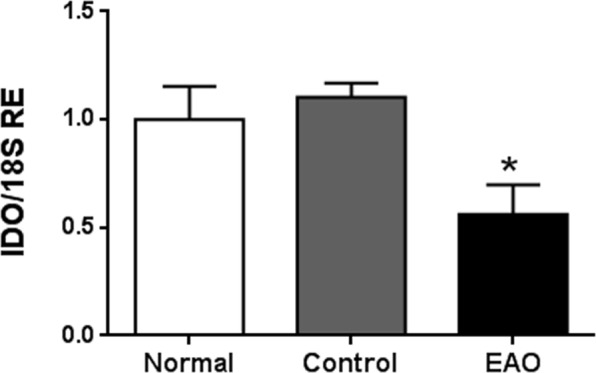
Testicular mRNA expression of IDO. Testicular mRNA expression of IDO was determined by qRT-PCR in testes from normal, control, and orchitis (EAO) rats. Results were normalized to *18S* rRNA housekeeping gene and expressed as fold change relative to testes of normal rats, to which a value of 1 was assigned. Values are mean ± SEM of relative expression units (RE) of 5 animals in each group. *P < 0.05.

To understand the relevance of IDO in testicular immune-privilege, we first compared IDO mRNA expression of N testis with IDO mRNA levels in testis from C and EAO groups. By qRT-PCR, we observed that testicular IDO expression was down regulated in rats with orchitis compared to C and N groups. Testis from C and N groups presented similar IDO mRNA expression levels (Fig. 1).

**Figure 2:**
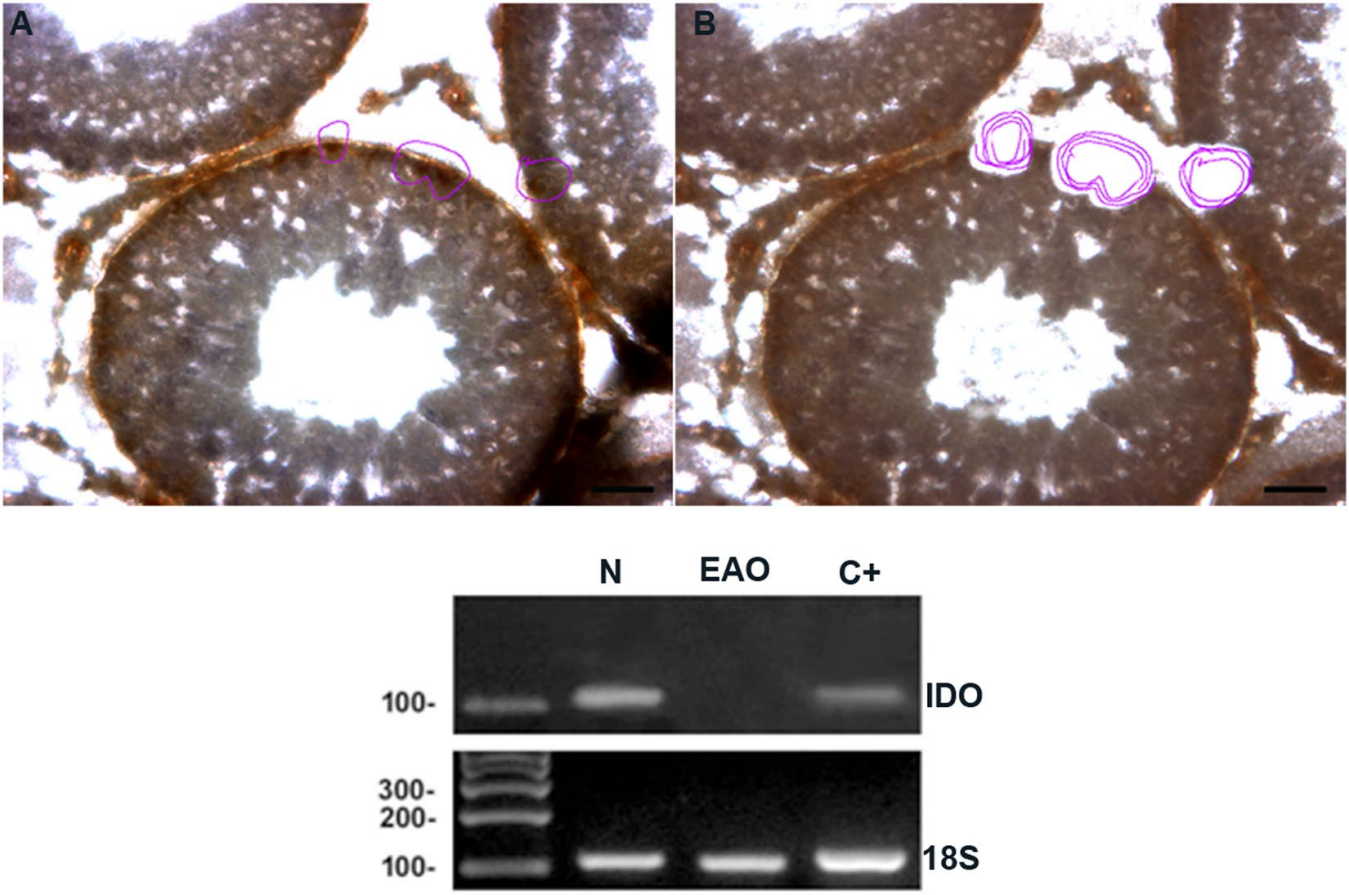
IDO is expressed in Sertoli cells. Vimentin-immunoreactive Sertoli cells from testicular sections of normal (N) and orchitis (EAO) rats were isolated by Laser Capture Microdissection (LCM). Representative microphotographs show the same section before (**A**) and after (**B**) LCM). Bar: 50 µm. Isolated Sertoli cells were subsequently used to evaluate the mRNA expression of *18S* rRNA (109 bp) and IDO (103 bp) by RT-PCR. Testis from N rats were used as positive control (C+).

Based on the fact that SC are considered the paradigmatic testicular somatic cells with an immune regulatory role, we evaluated IDO mRNA expression in SC isolated from N and EAO groups. LCM followed by RT-PCR analyses revealed visible PCR products only in SC from N testis (Fig. 2). In addition, expression of germ cell marker (VASA) was not seen in isolated SC (Fig. S1).

### Expression and localization of IDO and TDO

**Figure 3:**
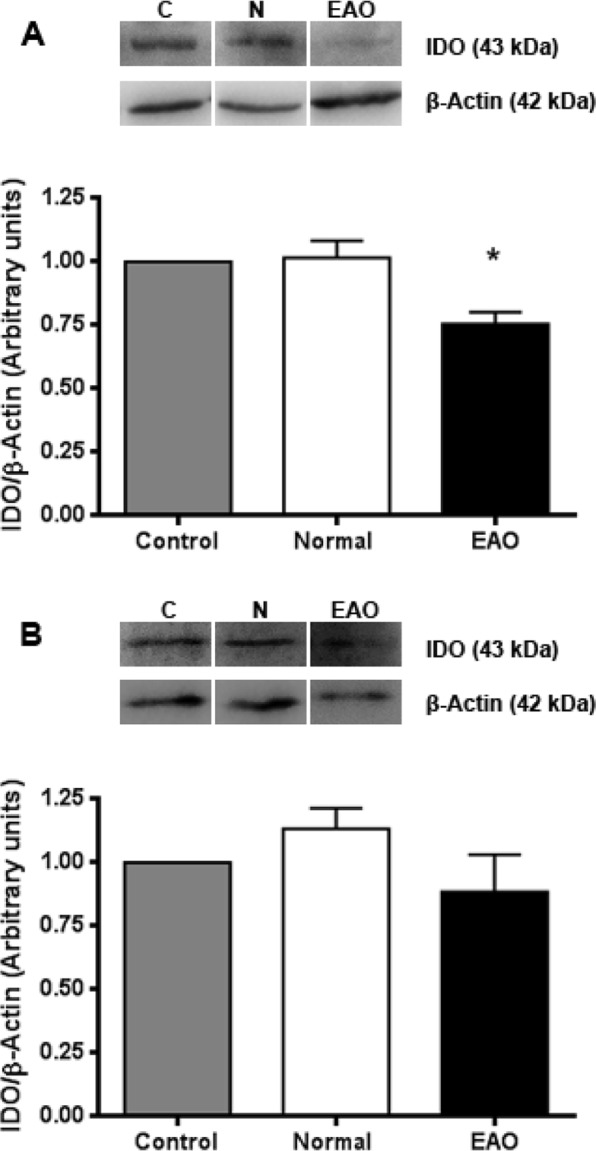
IDO1 is expressed in different testicular fractions. Expression of IDO by seminiferous tubules (**A**) and interstitial cells (**B**) of normal, control, and orchitis (EAO) rats was analyzed by Western blot. Each bar represents the mean ± SEM of 6 animals. The blots were cropped and the full-length blots are presented in the supplementary information (Fig. S2). *P < 0.05.

Protein IDO1 expression was evaluated by Western blot analysis in testicular ST and IC fractions from N, C, and EAO groups. IDO content in the ST fraction isolated from EAO rats was significantly lower compared to N and C rats (Figs 3A and S2A). No significant differences in IDO1 content were observed in the IC fraction in the different groups studied (Figs 3B and S2B).

**Figure 4:**
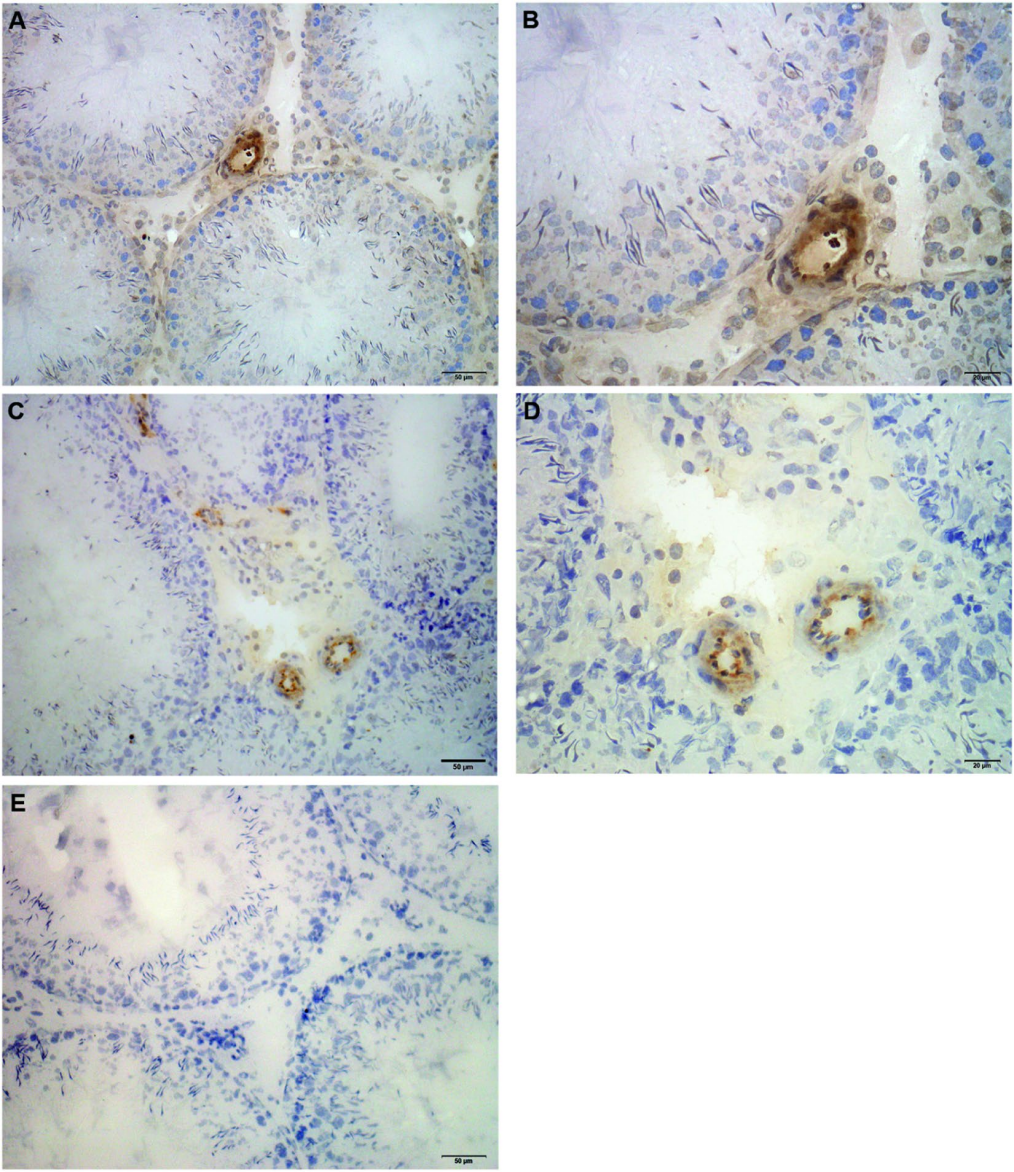
Immunohistochemistry of IDO1 in rat testis sections. Photomicrographs of adult testis cryostat sections immunostained with specific IDO antibody. Positive immunoreactivity can be observed in blood vessels of normal (**A**,**B**) and orchitis (EAO) (**C**,**D**) rats and also in mononuclear cells present in the testicular interstitium of EAO rats (**D**). Omission of primary anti-IDO1 antibody was used as a negative control (**E**).

In order to examine which cell populations of the testis might be contributing to IDO1 expression, immunohistochemistry was performed on testicular sections of N, C, and EAO groups. Intensity of IDO staining was similar in N (Fig. 4A–C (data not shown) rats. Positive staining was observed in the testicular interstitium of the three groups studied, localized mainly in the endothelium of blood vessels (Figs 4 and S3). In EAO rats mild IDO reactivity was also detected in mononuclear cells present in the testicular interstitium, identified as resident macrophages (ED2+ cells) and DC and inflammatory macrophages (ED1+ cells) (Fig. S3). Exceptionally, specific staining was detected in spermatid heads of ST (stages XI and XIV) from testes of N and C rats (data not shown).

**Figure 5:**
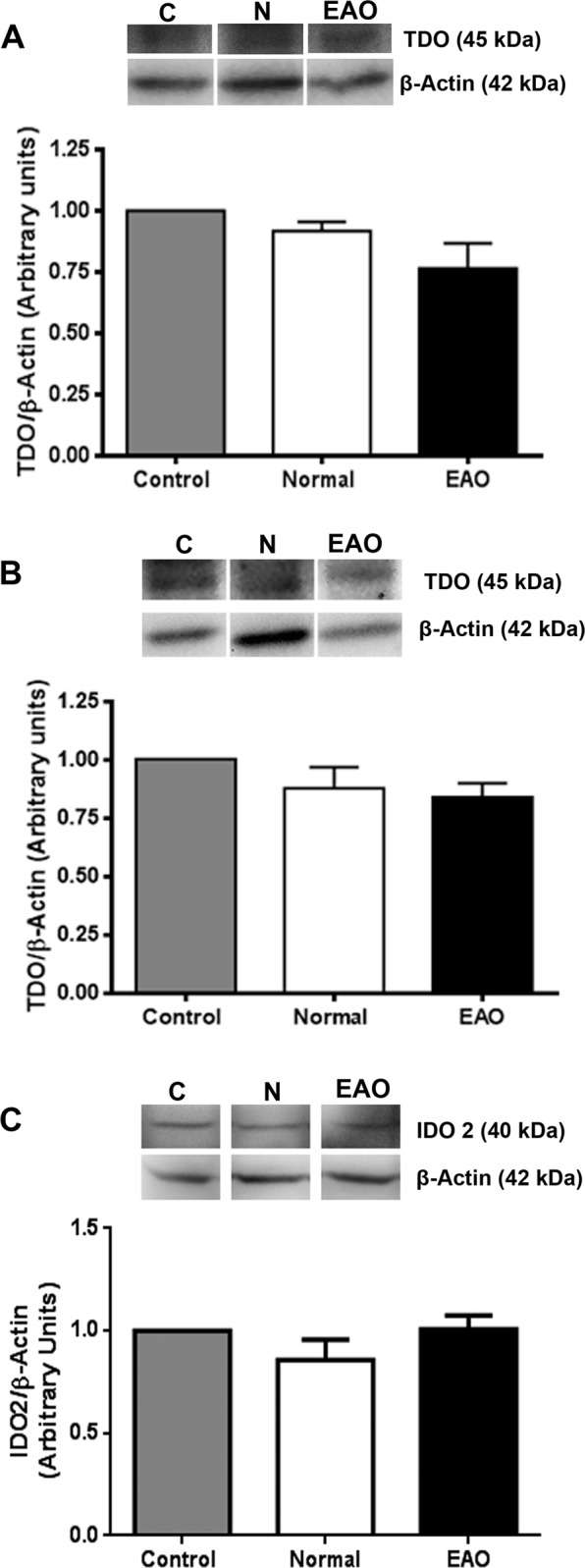
TDO and IDO2 expression in testis. Expression of TDO and IDO2 by seminiferous tubules (**A**), interstitial cells (**B**) and testis homogenate (**C**) of normal, control, and EAO rats was analyzed by Western blot. No changes in TDO and IDO2 expression were observed comparing the different groups. Each bar represents the mean ± SEM of 6 animals. The blots were cropped and the full-length blots are presented in the supplementary information (Fig. S4).

Considering that TDO and IDO2 also catalyze the initial step of tryptophan catabolism and TDO and IDO2 mRNA are expressed in testis^35,38^, we evaluated protein TDO and IDO2 expression. No significant differences in TDO and IDO2 content were detected by Western blot analysis in testis from N, C, and EAO groups (Figs 5 and S4).

**Figure 6:**
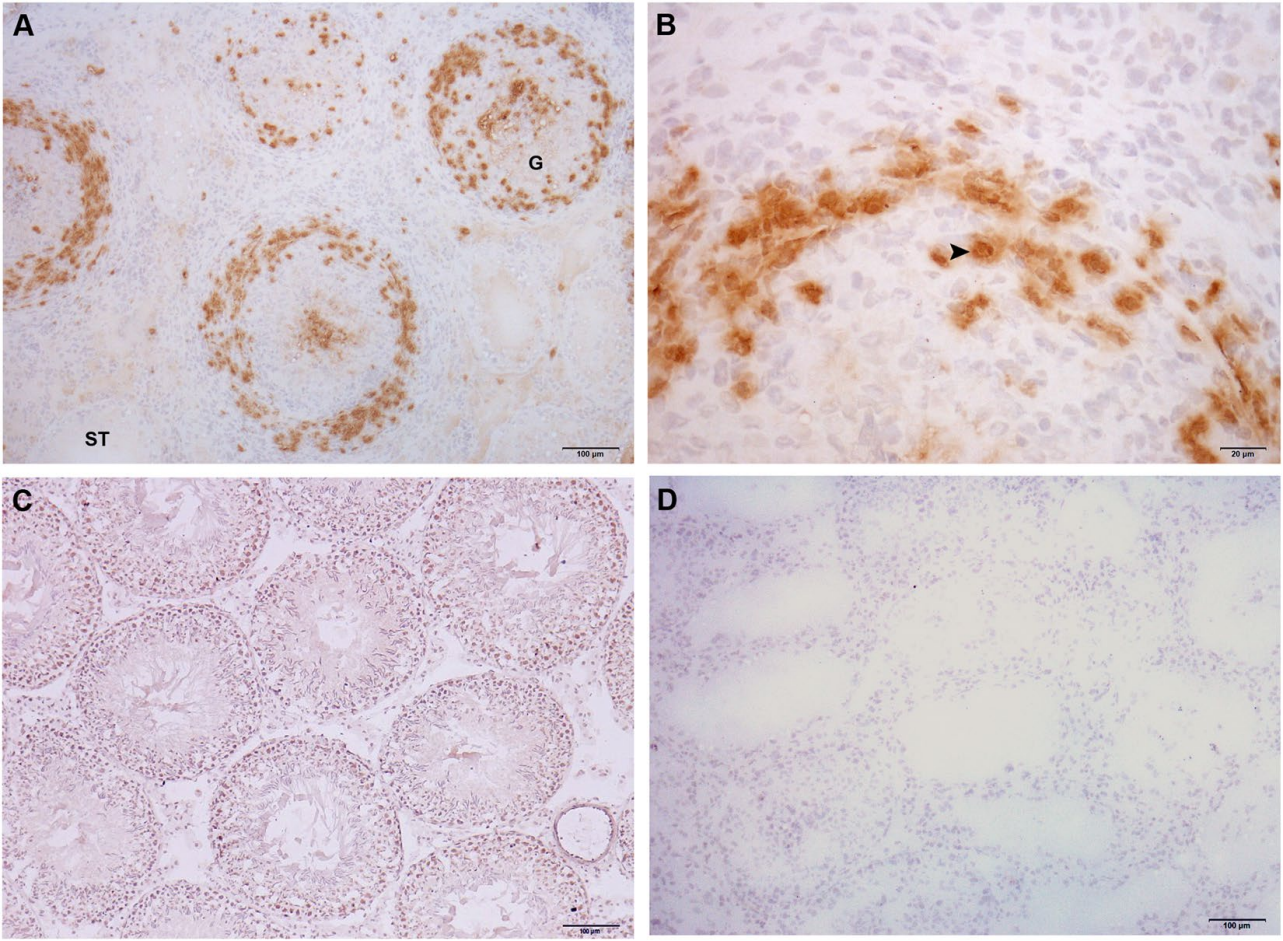
Immunohistochemistry of TDO in rat testis sections. Photomicrographs of adult testis cryostat sections immunostained with specific TDO antibody. Positive immunoreactivity can be observed in cells with macrophage/dendritic morphology (arrowhead) present in granulomas (G) and in the testicular interstitium of EAO rats (**A**,**B**). Negative reactivity can be observed in normal rat (**C**). Omission of primary anti-TDO antibody was used as a negative control (**D**). ST: seminiferous tubule.

TDO localization was evaluated by indirect immunoperoxidase in testicular sections of N, C, and EAO groups. TDO reactivity was detected in cells with macrophage/dendritic morphology present in granulomas and in testicular interstitium from EAO rats (Fig. 6A,B). No staining was observed in sections of N (Fig. 6C) and C rats (data not shown).

### IDO/TDO activity in testis

**Figure 7:**
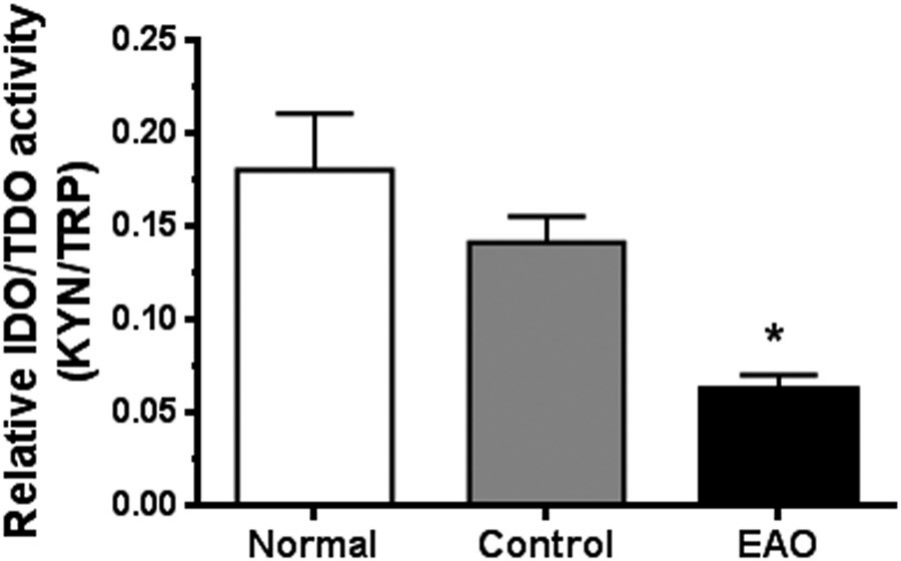
Evaluation of IDO/TDO activity. Testicular homogenates from EAO, control, and normal groups were used for measurement concentrations of kynurenine (Kyn) and tryptophan (Trp) by HPLC. IDO/TDO activity was expressed as the ratio of Kyn/Trp. Data are mean ± SEM of 6 animals. *P < 0.05.

IDO/TDO activity was assessed by HPLC to measure concentrations of Trp and Kyn in supernatants collected from testicular and LN homogenates. IDO activity (Kyn/Trp ratio) was reduced in testis of the EAO group compared to N and C groups (Fig. 7).

### *In vivo* inhibition of IDO increased severity of EAO

**Figure 8:**
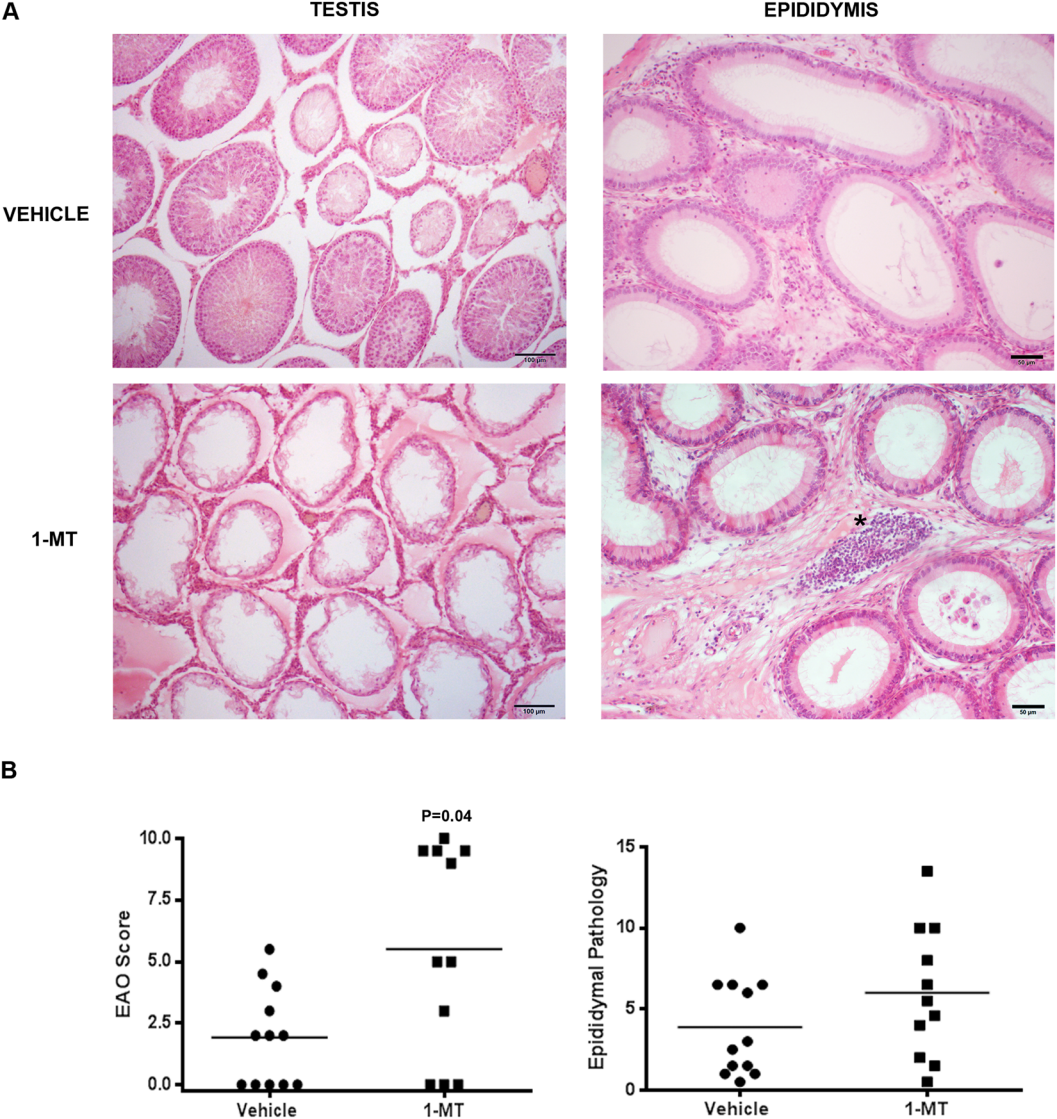
IDO blockade *in vivo*: administration of 1-Methyl-Tryptophan (1-MT). (**A**) Representative photomicrographs of paraffin testis and epididymis sections from immunized rats treated with vehicle or 1-MT. Rats from vehicle group presented focal testicular damage characterized by a moderate interstitial mononuclear cell infiltrate and several seminiferous tubules with different degrees of germ cell sloughing. In contrast, rats from 1-MT group presented severe orchitis with large areas of aspermatogenic seminiferous tubules showing only spermatogonia and Sertoli cells attached to the tubular wall. Epididymal histopathology of rats from 1-MT group compared to vehicle group shows similar depletion of spermatozoa and increase of inflammatory cell infiltrate (*). H&E. (**B**) Severity of orchitis and epididymitis is expressed as score mean ± SEM of vehicle and 1-MT group rats.

To elucidate the role of IDO in testicular immune regulation, we evaluated the impact of inhibiting IDO during EAO induction. As described in *Materials and Methods*, immunized rats were treated or not with a specific enzyme inhibitor 1-MT until euthanasia. Notably, rats treated with 1-MT during the immunization period developed orchitis with significantly increased severity (Fig. 8B). Testicular histopathology showed that vehicle group presented multifocal testicular damage characterized by mild infiltration of mononuclear cells and foci of ST with different degrees of germ cell sloughing. In contrast, most rats treated with 1-MT presented extended areas of severely damaged ST presenting aspermatogenesis (Fig. 8A).

Epididymis of EAO rats treated with 1-MT compared to EAO vehicle group showed a higher degree of inflammation represented by interstitial immune cell infiltrates and similar sperm depletion in the tubular lumen (Fig. 8A). However quantitative data of epididymal pathology were not statistically different (Fig. 8B).

Body weight of rats treated with 1-MT did not differ from that of untreated rats suggesting that no apparent detrimental effects were induced by IDO inhibition treatments (Body weight (g) mean ± SEM: 1-MT, 464.00 ± 9.73; vehicle group, 488.90 ± 9.40).

We subsequently investigated effects of IDO inhibition on T cell mediated immunity by analyzing DTH response to testicular antigens. Footpad challenge for DTH was tested in rats treated with 1-MT or vehicle 50 days after immunization. No significant differences in footpad swelling were observed between 1-MT and vehicle (Fig. S5). These results showed that both experimental groups reacted similarly to the antigen challenge independently of 1-MT treatment.

### Evaluation of IDO and TDO in lymph nodes

**Figure 9:**
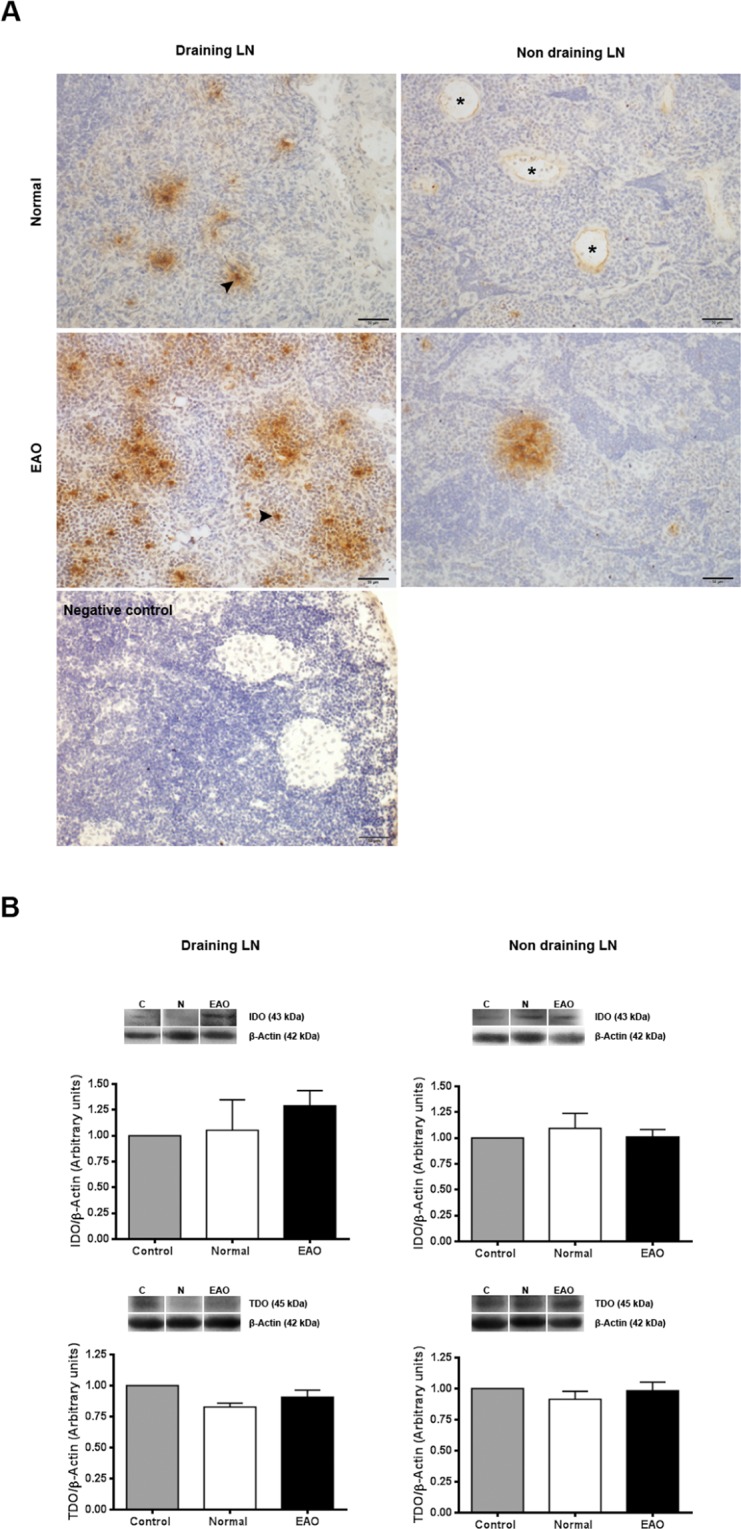
Evaluation of IDO1 and TDO in lymph nodes (LN). (**A**) Photomicrographs of testicular draining and non draining LN cryostat sections immunostained with specific IDO antibody. IDO immunoreactivity can be observed in endothelial cells of blood vessels (asterisk) and cells with macrophage/dendritic morphology (arrowhead). Note a higher number of positive IDO cells in draining LN from EAO rats. Omission of primary anti-IDO1 antibody was used as a negative control. (**B**) Expression of IDO and TDO in LN of normal, control, and EAO rats was analyzed by Western blot. Each bar represents the mean ± SEM of 6 animals. The blots were cropped and the full-length blots are presented in the supplementary information (Fig. S6).

Since draining LN reflect immune cell microenvironment of target organ, IDO1 and TDO expression was also evaluated in testicular draining LN and non-draining LN. Intense IDO staining was observed mainly in cells with macrophage/dendritic morphology present in the deep cortex and in the medullary cords of draining and non-draining LN. Endothelial cells from blood vessels also exhibited a slightly positive staining for IDO. The highest number of positive IDO cells was observed in draining LN of EAO rats. Also, a higher number of IDO positive cells were observed in draining LN compared to non-draining LN (Fig. 9A). LN of N rats presented similar IDO expression compared to rats in C group (data not shown).

No significant differences in IDO and TDO content were detected comparing N, C, and EAO rats in lysates of draining and non-draining LN (Figs 9B and S6).

**Figure 10:**
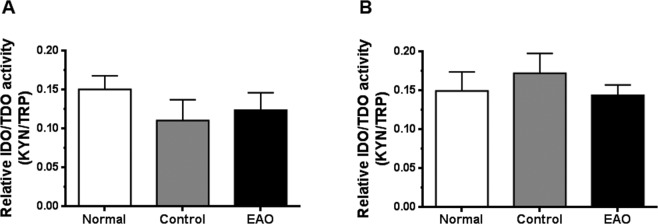
Evaluation of IDO/TDO activity in lymph nodes. Testicular draining (**A**) and non draining (**B**) lymph node homogenates from EAO, control, and normal groups were used for measurement concentrations of kynurenine (Kyn) and tryptophan (Trp) by HPLC. IDO/TDO activity was expressed as the ratio of Kyn/Trp. Data are mean ± SEM of 6 animals.

Regarding IDO/TDO activity in LN, no significant changes were detected in draining and non-draining LN of EAO rats compared to N and C groups (Fig. 10).

## Discussion

Although tryptophan catabolism into kynurenine by the IDO enzyme has been proposed as a putative mechanism in testicular immune tolerance, this possibility has never been evaluated in normal and pathological conditions in rats. This hypothesis stimulated us to evaluate the role of IDO in testis homeostasis and inflammation (autoimmune orchitis) in which immune privilege is disrupted. We evaluated the expression and activity of IDO/TDO in normal versus inflamed testis and assessed orchitis progression after treating rats immunized with sperm antigens and adjuvants with the IDO-specific inhibitor 1MT.

In the male reproductive tract, it has been described a null^35,36^ or negligible IDO mRNA and protein expression in the testis of mice^37^, contrasting with its high content in epididymis. More recently Jenabian *et al*.^38^ determined IDO1 and IDO2 mRNA in human testis of antiretroviral therapy-treated HIV- infected and non infected individuals. Our data show that rat testes with normal histopathology in the normal and control groups expressed IDO mRNA and IDO1 protein as analyzed by Western blot. By immunohistochemistry we observed that IDO1 + cells were found in the testicular interstitium mainly in endothelial cells, and also in mononuclear cells, as described for other organs^39,40^. The fact that we did not detect IDO1 in SC by immunohistochemistry but did identify IDO mRNA in isolated SC and IDO protein in seminiferous tubules by Western blot might be due to the different sensitivity of techniques. Prepuberal SC isolated from normal pig have been demonstrated to progressively produce IDO and prolong survival of co-grafts in different immunological settings^9,41^. As a relevant result, we observed IDO mRNA in SC from testis of normal untreated rats only but not in SC from rats with autoimmune orchitis. Notably, IDO mRNA and protein expression as well as IDO activity were reduced in testis from EAO rats, suggesting that testis inflammation is associated with downregulation of IDO expression and enzyme activity.

IFNγ produced in inflamed tissues is known to be potent IDO inducer because mammalian *ido1* gene promoters contain IFN-stimulated response elements^42^. We previously reported the increase of IFNγ in conditioned medium of testicular macrophages^27^ and in the number of IFNγ-producing CD4+ T cells in rats with orchitis^43^. However, present results do not reflect positive regulation of IDO by IFN. Also, *ido1* gene transcription may not result in enhanced IDO enzyme activity due to posttranslational controls, such as nitric oxide (NO)^44^. We may speculate that in EAO, NO–IDO interaction limits IDO function considering that testicular NO increases in rats with autoimmune orchitis^45^ and prevention of EAO development occurs by inhibition of the nitric oxide synthase-NO system^46^. Similarly, this interaction was also demonstrated in heart allografts in rats^47^ and in a model of collagen-induced arthritis^48^.

Under inflammatory conditions, IDO1 has been demonstrated to be subjected to proteasomal degradation in DCs, which is driven by high-level production of IL6, changing these cells from tolerogenic to immunogenic^49^. A high content of IL6 was previously demonstrated in autoimmune orchitis in rats^50,51^.

Although the number of IDO1+ cells in EAO draining LN was higher than in N draining- and non-draining LN, no significant changes in IDO1 expression and activity between N, C, and EAO groups were observed in draining and non-draining LN. In contrast, in a murine model of collagen-induced arthritis in which the tissue target is a non-immune privileged site IDO expression in LN was significantly increased after arthritis onset^48^. Both results reinforce our hypothesis that testicular IDO immune-suppression function occurs through a local mechanism.

In contrast to IDO1, no difference in testicular IDO2 content was detected comparing N, C, and EAO groups. It has been reported that deletion of the *Ido2* gene has no major impact on normal reproduction in the mouse^52^. Testicular TDO content was also similar among different groups studied. Notably, in few rats with severe orchitis; we observed TDO expression in macrophages and dendritic-like cells in the periphery of granulomae, which wall off immunogenic apoptotic germ cells from other structures of the tissue. By immunohistochemistry, Britan *et al*.^35^ detected that TDO protein was present both in the intertubular tissue and in late differentiating germ cells of adult mouse testis

Finally, to elucidate the role of IDO in testicular immune tolerance *in vivo*, we evaluated the impact of the specific IDO inhibitor 1-MT administered during the immunization period, demonstrating that IDO inhibition increased the severity of EAO. Therefore tryptophan metabolism could play a critical role in homeostatic control of testicular immune privilege. Since EAO is associated with epididymitis^26,32^, and the absence of IDO generates inflammation in the caput epididymis^53^, we also evaluated epididymis. IDO inhibition had no impact on epididymis histopathology. By analyzing *in vivo* DTH response to spermatic antigens, we showed that 1-MT treatment did not modify cell mediated immunity, thereby strengthening the hypothesis of a local mechanism of testicular IDO function as mentioned above in reference to LN results. Although they used a different experimental design of 1-MT administration, early results of Sakurai *et al*.^54^ and Kwidzinski *et al*.^55^ also showed that IDO inhibition exacerbates the clinical course of EAE, which supports a role for IDO in controlling T cell-mediated inflammation.

Although we did not explore the mechanisms of IDO function, its main functions are known to be depletion of tryptophan and production of bioactive tryptophan catabolites that would serve to suppress T-cell–mediated immune responses^42,56^.

Overall results highlight that IDO1 is an active enzyme expressed by rat testicular somatic cells which behaves as an immunosuppressor molecule able to modulate inflammatory immune response to spermatic antigens. Our *in vivo* findings provide new evidence that an IDO-based mechanism participates in testicular immune privilege.

## Supplementary information

Supplementary Info
