# Supplementary material for: Role of indoleamine 2,3-dioxygenase in testicular immune-privilege
Source: Sci Rep. 2019 Nov 4;9:15919. doi: 10.1038/s41598-019-52192-8 (PMC6828782; doi:10.1038/s41598-019-52192-8)
Supplement: Supplementary file 1 — Supplementary Info [file 41598_2019_52192_MOESM1_ESM.pdf]

Role of indoleamine 2,3-dioxygenase in testicular immune-privilege

Gisela S. Gualdoni, Patricia V. Jacobo, Cristian M. Sobarzo, Cecilia V. Pérez, María E. Matzkin, Christian Höcht, Mónica B. Frungieri, Marcelo Hill, Ignacio Anegon, Livia Lustig, Vanesa A. Guazzone

Supplementary Figure S1

Purity of isolated Sertoli cells after Laser Capture Microdissection

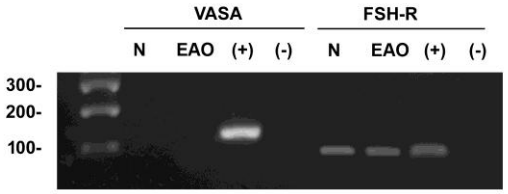

Vimentin-immunoreactive Sertoli cells (SC) from testicular sections of normal (N) and orchitis (EAO) rats were isolated by Laser Capture Microdissection. Isolated SC were subsequently used to evaluate the mRNA expression of VASA (germ cell specific marker, 127 bp) and FSH receptor (FSHR) (specific marker SC, 86 bp) by RT-PCR. Testes from N rats were used as positive control (+). (-): negative control.

Supplementary Figure S2

IDO is expressed in different testicular fractions

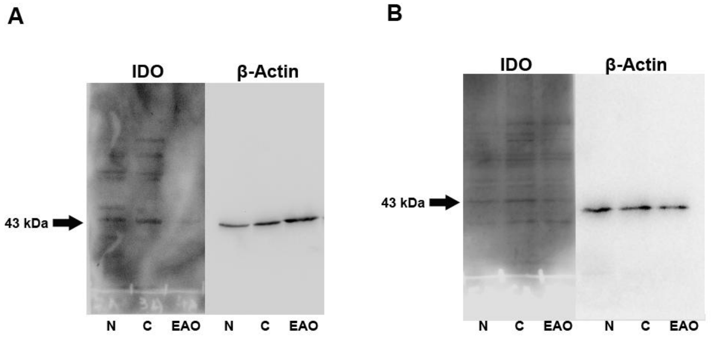

Full-lenght blots of IDO and β-Actin expression by seminiferous tubules (A) and interstitial cells (B) of normal (N), control (C) and orchitis (EAO) rats.

Supplementary Figure S3

IDO expression in ED1+ and ED2+ cells

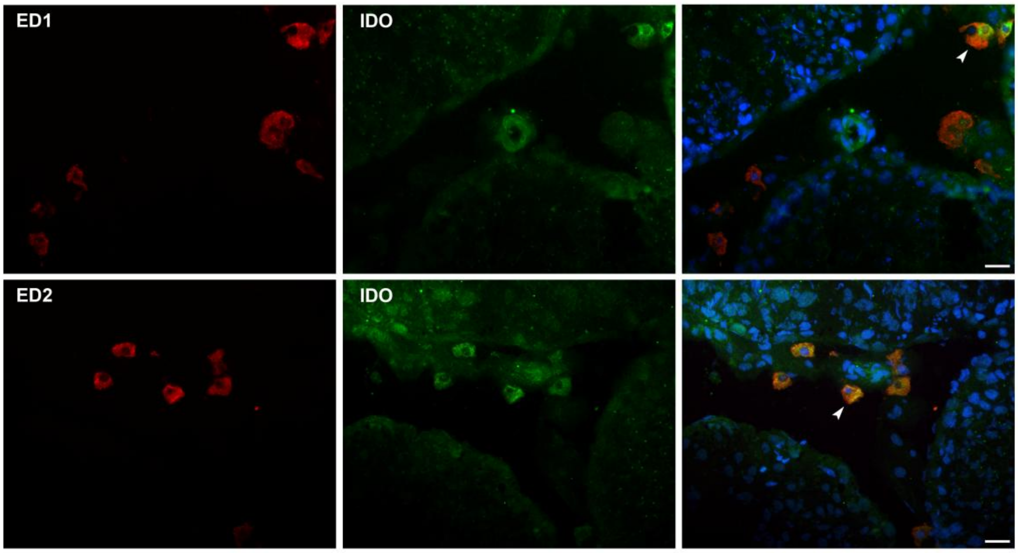

Expression of IDO in ED1+ or ED2+ cells in cryostat sections of testis from rats with orchitis. Secondary antibodies conjugated with fluorescein isothiocyanate or cyanine3 were used for IDO and ED1 and ED2 marker detection, respectively. All ED2+cells express IDO while not all ED1+ cells are IDO. Cell nuclei were stained with 4',6-diamidine-2-phenylindole (DAPI). Arrows indicate representative double positive cells. Bars: 20µm

IDO expression in endothelial cells

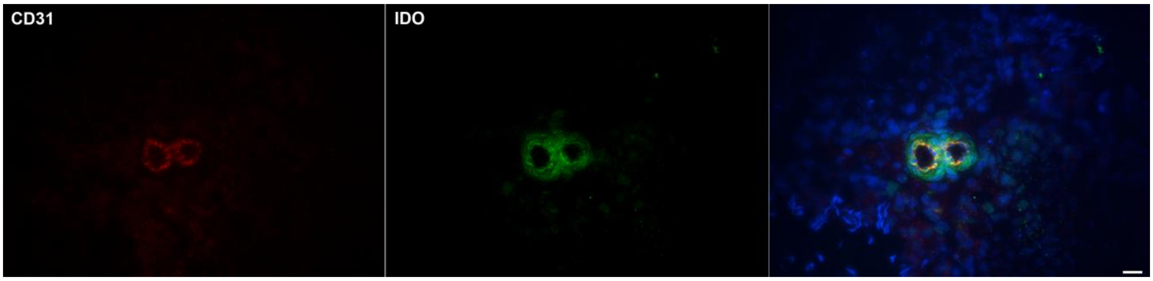

Expression of IDO in endothelial CD31+ cells in cryostat sections of testis from rats with orchitis. Secondary antibodies conjugated with fluorescein isothiocyanate or cyanine3 were used for IDO and CD31 marker detection, respectively. Cell nuclei were stained with 4',6-diamidine-2-phenylindole (DAPI). Bars: 20µm

Supplementary Figure S4

TDO expression in the testis

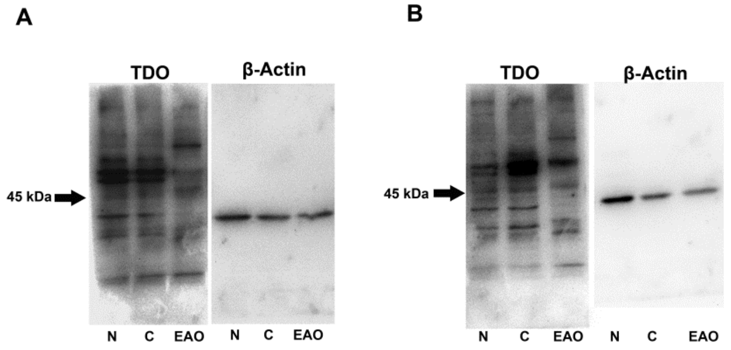

Full-lenght blots of TDO and β-Actin expression by seminiferous tubules (A) and interstitial cells (B) of normal (N), control (C) and orchitis (EAO) rats.

C **IDO 2 expression in the testis**

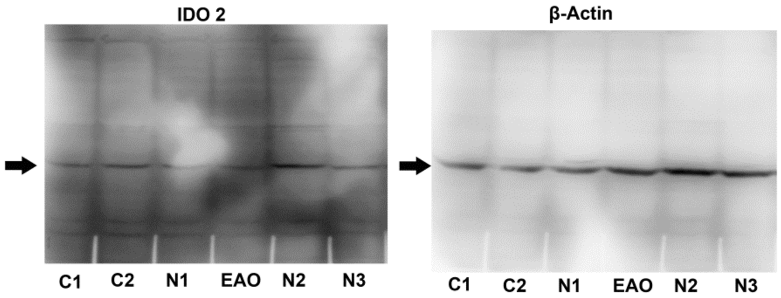

Full-lenght blots of IDO2 and β-Actin expression by testis of normal (N), control (C) and orchitis (EAO) rats.

Supplementary Figure S5

Delayed type hypersensitivity (DTH) is similar between 1-MT and vehicle groups

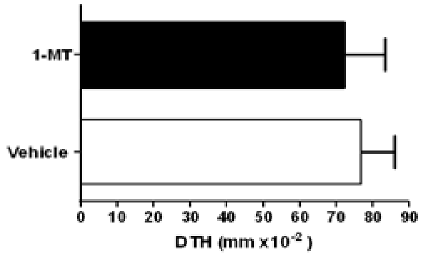

Rats immunized with TH and adjuvants were treated with 1-MT or untreated (vehicle) and tested for DTH to testicular antigens. Two days before euthanasia, rats were injected with TH into left footpad. The right footpad was injected with saline. Footpad swelling was measured after 48 h and results are expressed as the mean difference between the thickness of the TH-injected footpad and the thickness of saline-injected footpad ± SEM of 6 animals.

Supplementary Figure S6

Expression of IDO and TDO in LN

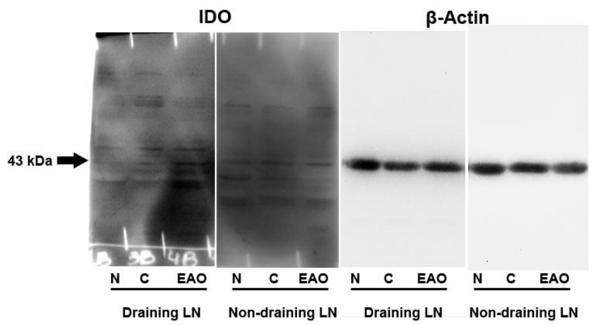

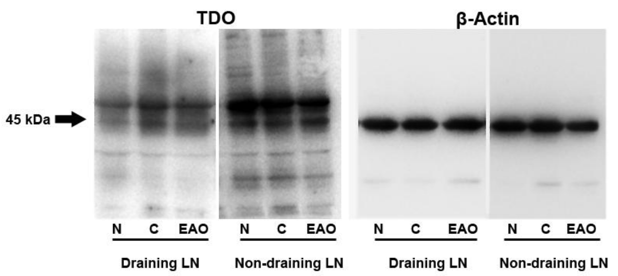

Full-lenght blots of IDO,TDO and β-Actin expression by draining and non-draining lymph nodes (LN) of normal (N), control (C) and orchitis (EAO) rats.
